# Development of an Innovative Glass/Stainless Steel/Polyamide Commingled Yarn for Fiber–Metal Hybrid Composites

**DOI:** 10.3390/ma16041668

**Published:** 2023-02-16

**Authors:** Anwar Abdkader, Muhammad Furqan Khurshid, Fathi Cherif, Mir Mohammad Badrul Hasan, Chokri Cherif

**Affiliations:** Faculty of Mechanical Science and Engineering, Institute of Textile Machinery and High-Performance Material Technology (ITM), Technische Universität Dresden, 01062 Dresden, Germany

**Keywords:** commingled yarn, hybrid yarn, air-jet texturing, glass, stainless steel, fiber–metal hybrid composite

## Abstract

Fiber–metal hybrid composites are widely used in high-tech industries due to their unique combination of mechanical, toughness and ductile properties. Currently, hybrid materials made of metals and high-performance fibers have been limited to layer-by-layer hybridization (fiber–metal laminates). However, layer-by-layer hybridization lacks in fiber to fiber mixing, resulting in poor inter-laminar interfaces. The objective of this paper was to establish the fundamental knowledge and application-related technological principles for the development and fabrication of air-textured commingled yarn composed of glass (GF), stainless steel (SS) and polyamide-6 (PA-6) filaments for fiber–metal hybrid composites. For this purpose, extensive conceptual, design and technological developments were carried out to develop a novel air-texturing nozzle that can produce an innovative metallic commingled yarn. The results show that an innovative metallic commingled yarn was developed using fiber–metal hybrid composites with a composite tensile strength of 700 ± 39 MPa and an E-modulus of 55 ± 7. This shows that the developed metallic commingled yarn is a suitable candidate for producing metal–fiber hybrid composites.

## 1. Introduction

Today’s megatrends in lightweight materials, material efficiency and CO_2_ reduction, as well as increased safety and performance requirements, demand innovative composite materials with defined tensile (stiffness and strength) and toughness properties. This can be achieved in particular by a combination of high-performance fibers, which have higher stiffness and strength, and traditional metal materials, which are characterized by good impact behavior, higher energy absorption and ductility. The hybridization of high-performance fibers with metal materials not only serves to achieve the desired mechanical properties but also to enhance the functional properties of the hybrids, e.g., providing an increase in electrical conductivity, thermal conductivity and temperature resistance [1,2,3,4,5]. Therefore, fiber hybrid composites have gained significant attention in recent years for developing components for the automotive, aerospace and aviation industries [6,7,8]. 

Currently, fiber hybrid composites composed of metals and high-performance fibers are limited, on the one hand, to intra-layer hybridization (yarn-by-yarn configuration) and, on the other hand, interlayer hybridization (layer-by-layer configuration). The layer-by-layer hybridization process is most common and has been widely established in the form of fiber–metal laminates (FML), where alternating thin layers of metal and fiber-reinforced plastics are combined. The main industrially adopted FMLs are GLARE (glass laminate aluminum-reinforced epoxy), CARALL (carbon-reinforced aluminum laminate) and ARALL (aramid-reinforced aluminum laminate) [9,10,11,12,13,14,15]. By using such hybrid components, positive hybrid effects can be achieved compared to when using pure fiber-reinforced plastics [16,17,18]. The improvement of the impact and crack resistance behavior and thus the improved damage tolerance of these hybrids is a good positive example [19,20,21] in addition to the improvement of the mechanical properties (static and dynamic), such as the increase in the stiffness, strength, fatigue strength [22,23,24,25,26,27,28] and damping, as well the electrical and magnetic properties [29,30,31]. Therefore, these fiber hybrid composites are widely used in fatigue-critical components that require high damage tolerance, good impact behavior and simple geometry [20,32,33]. Examples of their use include cockpits and the nose area of aircraft and helicopters, vertical stabilizers, cargo doors and seats, pipelines in the chemical industry and, especially, series applications in the automotive industry [34,35,36].

Despite this, the layer-by-layer configuration of metal and fiber reinforced plastics in FML also results in several disadvantages such as an insufficient mixing of components, very high manufacturing costs (e.g., five to ten times higher than those of aluminum) [36] and poor inter-laminar interfaces between the metal sheets and matrix material. Insufficient adhesion leads to premature failures in FML structures. This insufficiency is due to the geometrically limited interfaces between the metal sheet and the fiber-reinforced plastics and the associated low maximum transmissible shear stress [37,38]. To improve the adhesion properties or to increase the maximum shear stress at the interfaces, additional surface modification of the metal and/or the high-performance structure is necessary. However, surface modification based on plasma treatment does not eliminate delamination in these composites. Therefore, early component failure is not avoidable using this method. In order to exploit the performance potential of material hybridization, it is therefore necessary to develop innovative fiber hybrid composites where hybridization takes place at the fiber-to-fiber level (intra-yarn configuration). Fiber-to-fiber hybridization leads to improved interfacial bonding between the filaments and the matrix, which prevents delamination and cracking and significantly enhances the energy absorption capacity of the materials [39]. 

This fiber-to-fiber hybridization can be achieved through improving the homogeneous mixing of fibers in the structure. Undoubtedly, the commingling process based on air-jet texturing is a versatile technique that intermixes two or more filament yarns by intermingling the parallel arrangements of the infeed fibers. Generally, non-metallic high-performance and thermoplastic filament yarns are intermingled with a defined composition and mixing process for fiber hybrid composites. The commingling process based on air-jet texturing is divided into four sub-processes. These sub-processes are filament-feeding, opening, mixing and yarn winding. The air-jet commingling process delivers hybrid yarns with an improved mixing in the cross-section and an improved uniformity with a wide range of material combinations and desired yarn counts [40,41,42]. However, the air-jet commingling process is currently limited to the production of hybrid yarns from non-metallic fiber materials. Therefore, there is a strong need to modify the current air-jet commingling process to produce hybrid yarns based on high-density and bend-resistant metal filaments yarns for fiber–metal hybrid composites.

With this background, the aim of the current research was to develop an innovative commingled yarn based on metal, high-performance and thermoplastic filaments for fiber–hybrid composites. The design, construction and development of a novel air-texturing nozzle were conducted for the fabrication of the metal-based commingled yarn with an improved mixing and uniformity. The results proved that the developed air-texturing nozzle delivered innovative glass, stainless steel and polyamide-6 commingled yarn that is suitable for fiber–metal hybrid composites.

## 2. Materials and Methods

### 2.1. Development of Air-Texturing Nozzle for Processing Metal Filament Yarn

Air-jet texturing machines have been widely reported for fiber-to-fiber hybridization, and they produce commingled yarns with non-metallic high-performance (such as glass, carbon and aramid) and thermoplastic (polyamide and polypropylene) filament yarns for fiber-reinforced plastics. The air-jet nozzle is the central part of an air-texturing machine, and it performs the commingling operation and delivers hybrid yarns with improved mixing and uniformity [43,44,45,46,47]. However, the current air-jet nozzles (such as Taslan, Du Pont, Hemajet and Heberlein) are unsuitable for processing bend-resistant and dense metal filaments. To overcome this problem, the design, construction and development of a new air-jet nozzle for the development of innovative hybrid yarn composed of metal, high-performance and thermoplastic filaments was the focus of this research.

In order to achieve a better spreading and intermixing of the metal, high-performance and thermoplastic fibers with low damage, an air-texturing nozzle based on Hemajet was analyzed experimentally by considering the special properties of the metal fiber (e.g., high stiffness, high density and bend resistance). For this purpose, two target variables were defined:

1. In order to avoid turbulence or perpendicular flow in the compressed air against the fiber guiding direction, the flow generated by air pressure needed to be as parallel as possible to the fiber guiding direction at the first contact with the fibers.
2. In order to overcome the high bending stiffness of the heavy metal filament yarn, the flow velocity needed to be at a maximum before reaching the outlet cross section of the nozzle unit. The decisive parameters for this were the angle and profile of the air channel as well as the profile of the yarn outlet in the nozzle design.

To achieve the above-mentioned objectives, the following conditions were assumed for the simulation:

➢ The filament feeding needed to not negatively influence the airflow.
➢ The supersonic flow created by the Laval element needed to be guided along the airflow in such a way that the filaments were moved in a preferential direction.
➢ The filaments needed to preferably be deflected with large radii transverse to the yarn direction. Furthermore, the filaments needed to be deflected in such a way that the different types of fibers were homogeneously distributed in the yarn cross-section.

**Figure 1:**
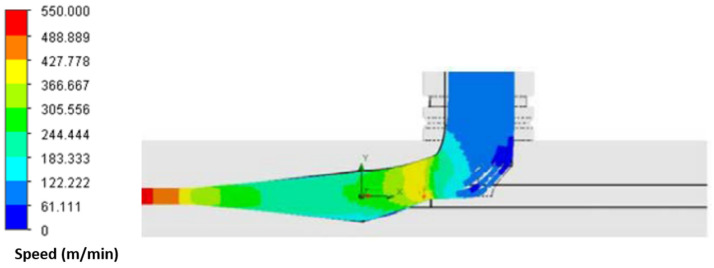
Optimized novel air-jet nozzle geometry with air flow profile.

The design and development of the novel air-texturing nozzle unit, referred as the ITM-HiMeJET nozzle, was carried out with the SOLIDWORKS software 2020 version 28 and, particularly, with the SOLIDWORKS Flow Simulation extension. For this purpose, various shapes of the airflow path and nozzle geometries were analyzed to identify optimum design. In addition to this, the diameter of the nozzle, flow velocity, boundary conditions and thermodynamic parameters were optimized. Figure 1 shows the optimized airflow profile of the novel air-texturing nozzle as a function of the nozzle geometry. The optimized geometry delivered an effective airflow for the mixing of the metal and high-performance filaments, as shown in Figure 1.

**Figure 2:**
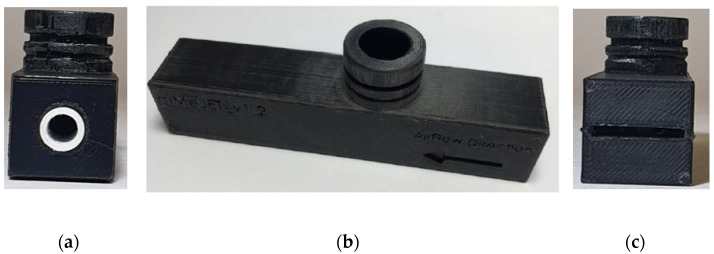
Novel air-texturing nozzle produced with additive manufacturing technique: (**a**) feed section, (**b**) main module and (**c**) delivery section.

Based on the simulation, the optimized design of the novel air-texturing nozzle was constructed by using an additive manufacturing technique (3D printer Objet30 Prime from Stratasys, Switzerland). In order to achieve this, continuous PLA filaments (Prusa PLA Jet Black, Prague) with a diameter of 1.75 ± 0.02 mm were used as the construction material. The 3D printer precisely developed the air-texturing nozzle, as shown in Figure 2. 

### 2.2. Development of Innovative Hybrid Yarns

**Figure 3:**
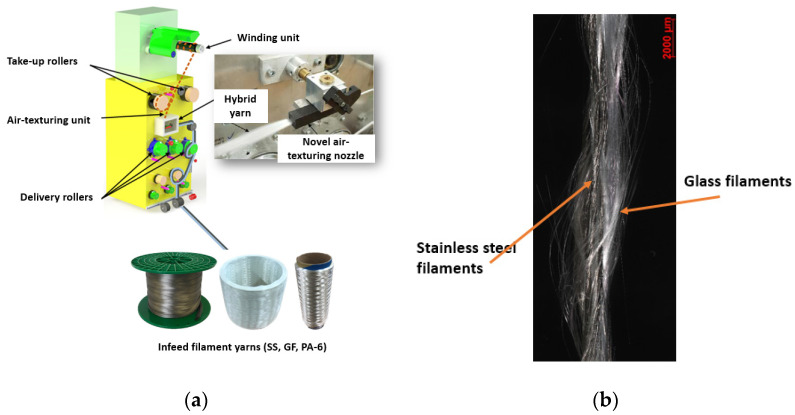
Novel air-texturing nozzle: (**a**) implementation and (**b**) commingling yarn.

In this phase, the new air-jet nozzle was installed on an air-jet texturing machine (Stahle-Eltex GmbH, Weil am Rhein, Germany) to study the influence of the nozzle design on the commingling behavior. For this purpose, the air pressure, feeding speed, take-off speed and overfeeding percentage were adjusted for the development of the innovative commingled yarn. The investigation revealed that an air pressure of 3 bar and an overfeed of 5% were the optimum parameters for producing the innovative hybrid yarn based on metal, high-performance and thermoplastic filaments. It was also observed during the experimentation that slippage of the metal filaments occurred on the feed godet, especially when a high overfeed was used. To reduce the slippage, a polyolefin shrink sleeve was used on the godet. The results showed that hybrid yarn based on the novel air-texturing nozzle had a very high degree of opening and mixing as well as low fiber damage, as shown in Figure 3.

For the development of the innovative hybrid yarns, stainless-steel (SS) filament yarn from NV Bekaert SA, Zwevegem, Belgium, glass filament yarn (GF) from the company P-D Glas-Seiden GmbH, Oschatz, Germany, and polyamide filament yarn (PA-6) from PHP Fibers GmbH, Erlenbach, Germany, were purchased. Furthermore, hybrid yarns based on glass, stainless-steel and polyamide filament yarn with two different compositions were produced for the fiber–metal hybrid composites. The details of the raw materials used for the development of the innovative GF/SS/PA-6 commingled yarn are given in Table 1.

### 2.3. Development of Unidirectional Composites

**Figure 4:**
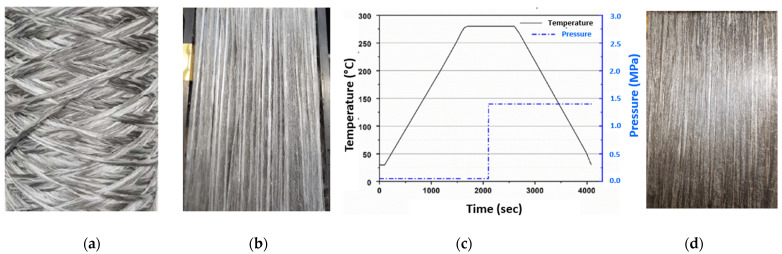
Composite development: (**a**) innovative commingled yarns, (**b**) unidirectional prepreg, (**c**) consolidation cycle and (**d**) fiber–metal hybrid composite.

For the investigation of the mechanical properties, unidirectional composites were produced from these innovative GF/SS/PA-6 commingled yarns. For this purpose, the commingled yarns were wound onto a winding frame. Subsequently, the unidirectional composite sheets were produced using a P300 PV laboratory hot-press machine (Collin, Germany). The consolidation was carried out at 280 °C and a pressure of 1.40 MPa. Figure 4 shows the innovative hybrid yarns, unidirectional prepreg, consolidation cycle and fiber–metal hybrid composites. 

### 2.4. Characterization of Fibers, Hybrid Yarns and Composites

The single-fiber strength of the infeed filaments (SS, GF, PA-6) was measured with a Favimat single-fiber tensile testing machine from Textechno Herbert Stein GmbH & Co. KG, Mönchengladbach, Germany. The surface morphology of the metal filaments was taken by using a Quanta scanning electron microscope from Thermo Fisher Scientific Inc., Waltham, MA, USA. Tensile property tests of the innovative commingled yarns were carried out according to the DIN EN ISO 3341 standard test method on a Zwick type Z/100 (Zwick GmbH and Co., Ulm, Germany) tensile testing machine. For this purpose, yarn samples of a 250 mm length were used. The test velocity was set to 100 mm/min, and the initial load was kept at 0.5 cN/tex. For the tensile properties of the composites, composite specimens in the 0° direction with a length of 250 mm, a width of 25 mm and a thickness of 2 mm were cut and subjected to the same Zwick type Z/100 (Zwick GmbH and Co., Germany) tensile testing machine according to the DIN EN ISO 527-5/A/2 standard test method [48]. 

## 3. Results and Discussion

The tensile response of unidirectional hybrid composites depends on the tensile properties of their individual constituents. Therefore, the tensile properties of glass, stainless-steel and polyamide filaments were tested and are presented in the first part of this section. Subsequently, these properties were correlated with the mechanical response of the hybrid yarns and the corresponding fiber–metal hybrid composites in a later section.

### 3.1. Properties of Metal and Glass Filaments

#### 3.1.1. Surface Morphology and Topography

**Figure 5:**
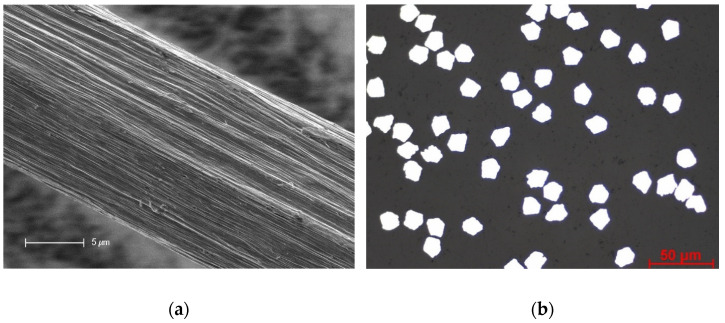
Microscopic view of stainless-steel material: (**a**) longitudinal view and (**b**) cross-sectional view.

Figure 5 shows the longitudinal and cross-sectional view of the stainless-steel fibers observed under a microscope. In the longitudinal view, the surface of the stainless steel consisted of continuous groves, which were parallel to the fiber direction and were a result of the multi-stage stretching zones during the drawing process of the thin wire during production. The results of the geometric cross-sectional analysis of the stainless-steel filaments showed that the cross-section of the stainless steel was not circular but irregular, ranging from a triangular to a trapezoidal shape. In contrast, it has been well established that glass fibers are circular in their cross-section and have a smooth surface [49]. 

**Figure 6:**
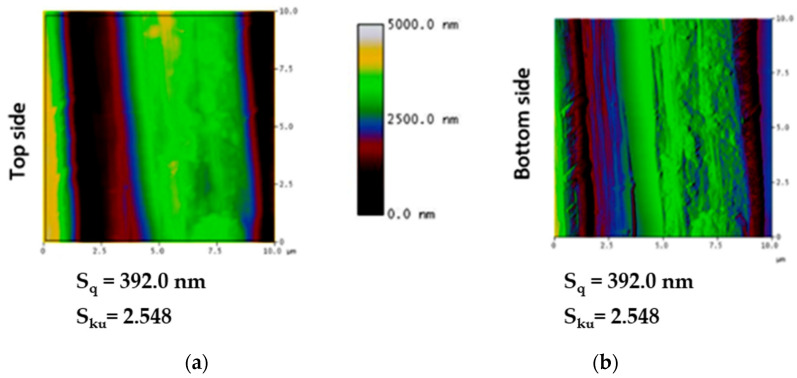
Topography of the stainless-steel filaments: (**a**) top surface and (**b**) bottom surface.

The results of the surface topography analysis of the stainless-steel filaments, quantified with atomic force microscopy, are presented in Figure 6. The surface topography was measured in terms of height parameters, including the mean-square height of the scale-limited surface (S_q_) and the kurtosis of the scale-limited surface (S_ku_). These height parameters determine whether a material’s surface is either smooth or rough. The results showed that the S_q_ value of the top and bottom sides of the stainless-steel filaments were 392.090 nm and 718.193 nm, respectively. However, the S_ku_ values from the top and bottom sides were 2.548 and 2.821, respectively. These high values of S_q_ reflect that the stainless-steel filaments exhibited a uniform surface.

#### 3.1.2. Tensile Properties

The tensile properties of individual components play a critical role in determining the tensile properties of high-performance composites. In this case, two high-performance fibers, glass and stainless-steel filaments, were used in this study. Their tensile properties determined the yarn and composite response under the loading conditions. Therefore, the single-fiber strengths of the glass and stainless-steel filaments are presented in this section.

**Figure 7:**
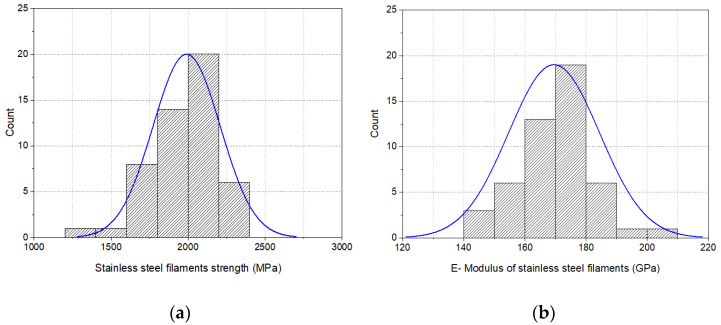
Tensile properties of stainless-steel filaments: (**a**) strength distribution curve and (**b**) E-modulus distribution curve.

Figure 7a,b present the distribution curve of the tensile properties of the stainless-steel filaments. The results of the single-fiber strength test show that the stainless-steel filaments used in this study had an average tensile strength of 2015 ± 222 MPa and an E-modulus of 170 ± 15 GPa. The elongation at break of the stainless-steel filaments lay in the range of 1.5 ± 0.1%. The low elongation behavior confirmed that the used stainless-steel filaments were highly stretched during their production, particularly in the multi-stage drawing and drafting zones and, therefore, possessed a very high tensile strength and E-modulus.

**Figure 8:**
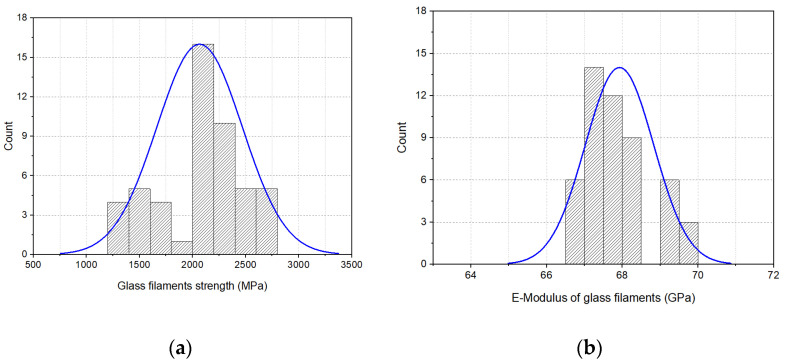
Tensile properties of glass filaments: (**a**) strength distribution curve and (**b**) E-modulus distribution curve.

The second main constituent employed in this study to develop the fiber–metal hybrid structures was the glass filaments. The distribution curves of the tensile properties of the glass filaments are presented in Figure 8a,b. The glass filaments had an average tensile strength of 2058 ± 224 MPa, an E-modulus of 68 ± 1.5 GPa and an elongation at break of 3.4 ± 1.7%. 

In the case of the polyamide filaments, their tensile strength, E-modulus and elongation at break were 262 ± 35 MPa, 0.9 ± 0.1 GPa and 175 ± 30%, respectively. However, the tensile properties of the polyamide did not remain the same after the consolidation process. Therefore, the ensile properties were taken by developing a polyamide composite (in a consolidation form). In the consolidation form, the tensile strength, E-modulus and elongation at break were 40 ± 2.6 MPa, 2.62 ± 0.1 GPa and 75 ± 3.4%, respectively [50]. 

### 3.2. Properties of GF/Stainless-Steel/PA6 Hybrid Yarns

**Figure 9:**
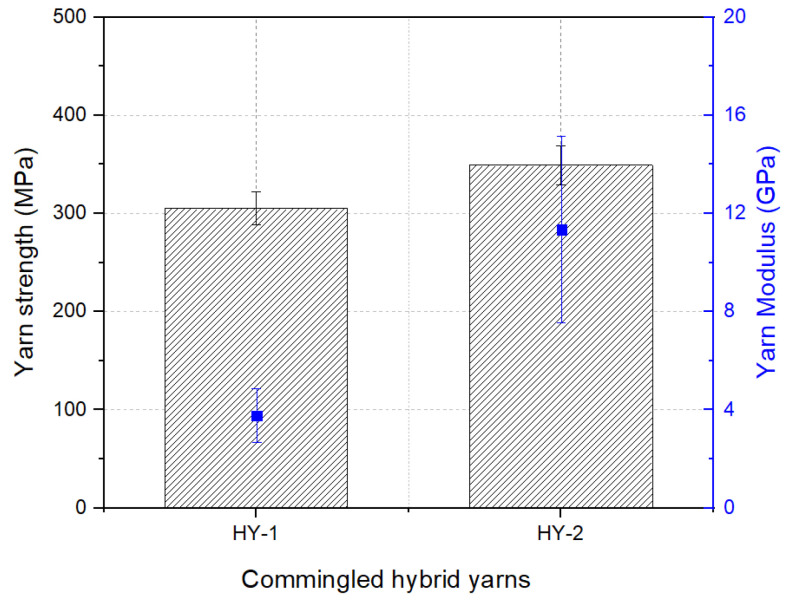
Tensile properties of GF/stainless-steel/PA-6 commingled yarns.

Commingled yarns based on GF/stainless-steel/PA6 filaments were developed with two different material compositions as reported earlier. The tensile response of the HY-2 commingled yarn made of these filaments was relatively higher compared to the HY-1 commingled yarn, as shown in Figure 9. This was compared with the composition and tensile properties of the stainless steel and glass fibers in the yarn structure. In both the commingled yarns, the percentage of the high-performance materials (stainless steel and glass) in the yarn structure was about 44% (by volume). 

However, the composition of low-elongation (LE) fibers, which were composed of stainless-steel fibers, was higher in the HY-2 commingled hybrid yarn compared to the HY-1 commingled yarn. In hybrid structures, it has been well established that the mechanical response of a hybrid structure depends on its composition of low-elongation fibers rather than high-elongation (HE) fibers. Generally, a higher content of LE fibers delivers a higher tensile strength in the structure of hybrid materials [44]. Furthermore, the tensile strengths of the LE fibers (stainless-steel filaments) and HE fibers (glass filaments) utilized in this research lay in a similar range (approximately 2000 MPa), as presented earlier. Therefore, the HY-2 commingled hybrid yarn showed a higher tensile response compared to the HY-1 commingled yarn.

### 3.3. Properties of Composites Based on GF/Stainless-Steel/PA6 Commingled Yarn

**Figure 10:**
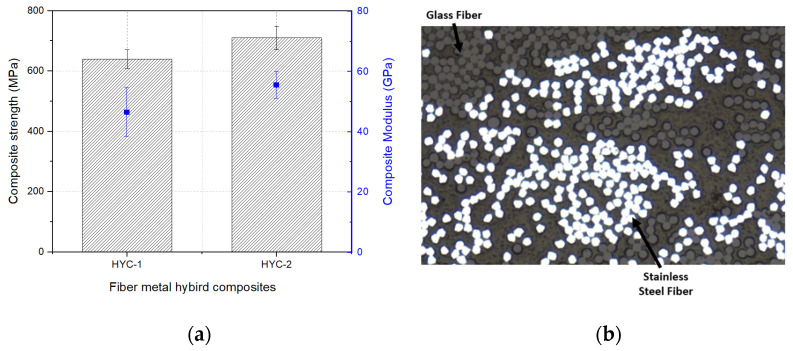
GF/stainless-steel/PA6 hybrid composites: (**a**) tensile properties and (**b**) cross-sectional view of HYC-2 hybrid composite.

The tensile properties of the fiber–metal composites developed from the two hybrid yarns (HY-1 and HY-2) are referred to as HYC-1 and HYC-2. In both the hybrid yarns, their compositions of stainless-steel fibers were 18 and 22 percent, respectively. However, the polyamide volume fraction remained constant in both the fiber–metal composites. The results of the composites, shown in Figure 10a, highlighted that the composite developed from the HY-1 hybrid yarn had a lower tensile modulus and strength as compared to the composite developed from the HY-2 hybrid yarns. This was correlated with the composition of LE fibers present in the composite structure [51]. 

In the case of the fiber–metal hybrid composite developed from commingled hybrid yarns, the stainless-steel fiber was the LE fiber, as reported in the previous section. Therefore, failure of the stainless-steel fibers occurred earlier in the composite structure and was followed by failure of the glass and polyamide materials. The reason was that the stainless-steel fibers utilized in this study had a low elongation at break (1.5%) compared to the glass and polyamide materials. Furthermore, it has been well established that the tensile properties of hybrid composites are significantly determined by the content of LE fibers present in the hybrid composites. Consequently, the fiber–metal hybrid composite fabricated from the HY-2 commingled hybrid yarn showed higher tensile properties compared to the composite fabricated from the HY-1 hybrid yarn.

In order to estimate the tensile properties of hybrid composite, the rule of hybrid mixtures (RoHM) is widely utilized in the literature [48]. Based on this rule, the tensile modulus and strength of hybrid composites depends on the tensile modulus, tensile strength and fiber volume content of their individual constituents. The equations of the rule of hybrid mixtures are expressed in Equations (1) and (2).

(1) $$E_{11}=V_{f1}\cdot \;E_{11}^{f1}\;+V_{f2}\cdot \;E_{11}^{f2}\;+V_{m}\cdot \;E^{m}$$

(2) $$E_{11}=V_{f1}\cdot \;E_{11}^{f1}+V_{f2}\cdot \;E_{11}^{f2}+V_{m}\cdot \;E^{m}$$

Here, $E_{11}^{f1},\;E_{11}^{f2}\;and\;E^{m}$ refer to the tensile modulus of the first fiber, second fiber and matrix, while $\sigma_{11}^{f1},\;\sigma_{11}^{f2}\;and\;\sigma^{m}$ represent the tensile strength of the first fiber, second fiber and matrix. Furthermore, $\;V_{f1}$, $\;V_{f2}\;and\;V_{m}$ denote the fiber volume content of the first fiber, second fiber and matrix. A recent study conducted a finite element analysis to predict the mechanical properties of hybrid composites [52]. This study disclosed that the tensile modulus and strength of the hybrid composites were in a good agreement with Equations (3) and (4). These equations were modified from the rule of hybrid mixtures (MRoHM).

(3) $$E_{11}=V_{f1}\cdot \;E_{11}^{f1}+V_{f2}\cdot \;E_{11}^{f2}+V_{m}\cdot E^{m}$$

(4) $$\sigma_{11}=E_{11}\cdot \;e_{c}^{\left(+\right)}\;$$

Using Equations (3) and (4), the tensile modulus of a hybrid composite can be estimated, similarly to the rule of hybrid mixtures. However, the tensile strength is a function of the modulus of a hybrid composite and the failure of a component with the least strain. In the case of the Glass/SS/PA-6 hybrid composite, the least-strain component present in the structure was the stainless-steel filament. Therefore, the tensile strength of the hybrid composites was a function of the tensile modulus of the hybrid composite and the strain of the stainless-steel material. Table 2 presents the results of the experimental and estimated tensile properties of the Glass/SS/PA-6 hybrid composites. The experimental results of the tensile modulus provided a very good estimation from the modified rule of hybrid mixtures (MRoHM). In the case of the tensile strength, an error of around 12 to 15 percent was observed from the experimental results. This indicates that further investigations are required to optimize the air-texturing and consolidation process to develop fiber–metal hybrid composites.

## 4. Conclusions

In this paper, innovative commingled yarns based on stainless-steel, glass and polyamide filament yarns for fiber–metal hybrid composites were developed. For this purpose, the design, construction and development of a novel air-texturing nozzle were implemented using an air-texturing machine to develop and produce the innovative commingled hybrid yarn structures. The results showed that the newly designed air-texturing nozzle delivered metal commingled hybrid yarns with improved fiber-to-fiber mixing and uniformity. The unidirectional fiber–metal hybrid composites developed from these innovative commingled hybrid yarns showed promising tensile properties (700 ± 39 MPa and E-modulus of 55 ± 7) compared to theoretical estimates. Based on this novel air-texturing nozzle, it is possible to develop different commingled yarns by altering the type of metal filaments (e.g., stainless steel, copper and aluminum), the type of high-performance filament (e.g., carbon and aramid) and the type of matrix filament (e.g., polyester, polypropylene, polyether ether ketone and polybutylene succinate) with different compositions for fiber–metal hybrid composites.

## Figures and Tables

**Table 1:** Properties of the raw materials.

Properties	Unit	Commingled Yarn	
		HY-1	HY-2
Glass filament yarn linear density/number of filament yarns	tex/No.	200/1	200/1
Stainless-steel filament yarn linear density/ number of filament yarns	tex/No.	105/4	105/5
Polyamide filament yarn linear density/ number of filament yarns	tex/No.	47/4	47/5
GF/SS volume fraction	%	26/18	22/22
PA-6 volume fraction	%	56	56
Commingled yarn linear density	tex	835	1094

**Table 2:** Experimental and estimated results of Glass/SS/PA-6 hybrid composites.

Composite	$E_{11}\;(\mathrm{GPa})$				$\sigma_{11}\;(\mathrm{MPa})$	
	Experimental	MRoHM	Error	Experimental	MRoHM	Error
HCY-1	47 ± 6	49.9	6.17	640 ± 25	748.5	14.4
HCY-2	55 ± 7	53.5	2.8	710 ± 39	802.5	12.9

## Data Availability

The data underlying this article will be shared on reasonable request from the corresponding author.
